# Early use of intrapartum intra-aortic balloon pump support for haemodynamic stabilization of peripartum and anthracycline-induced cardiomyopathy: a case report

**DOI:** 10.1093/ehjcr/ytae033

**Published:** 2024-01-23

**Authors:** Karthic Chandran, Donald Quimby, Hiram G Bezerra, Daniela Crousillat

**Affiliations:** Division of Cardiovascular Sciences, Department of Medicine, University of South Florida, 2 Tampa General Circle, Tampa, FL 33606, USA; Division of Cardiovascular Sciences, Department of Medicine, University of South Florida, 2 Tampa General Circle, Tampa, FL 33606, USA; Division of Cardiovascular Sciences, Department of Medicine, University of South Florida, 2 Tampa General Circle, Tampa, FL 33606, USA; Tampa General Hospital Heart and Vascular Institute, Interventional Cardiology Center of Excellence, 2 Tampa General Circle, Tampa, FL 33606, USA; Division of Cardiovascular Sciences, Department of Medicine, University of South Florida, 2 Tampa General Circle, Tampa, FL 33606, USA; Tampa General Hospital Heart and Vascular Institute, Women’s Heart and Cardio Obstetrics Program, 2 Tampa General Circle, Tampa, FL 33606, USA

**Keywords:** Cardio-obstetrics, Pregnancy, Heart failure, Peripartum cardiomyopathy, Cardiogenic shock, Intra-aortic balloon pump, Case report

## Abstract

**Background:**

Prior exposure to cardiotoxic cancer therapies has been associated with an increased risk of peripartum cardiomyopathy (PPCM). The management of PPCM in this population remains a clinical challenge. Few studies have explored the use of mechanical circulatory support in PPCM. We present a case of early implementation of intra-aortic balloon pump (IABP) therapy for acute stabilization and intrapartum support of PPCM.

**Case summary:**

A 36-year-old G4P2103 (4th pregnancy, two full-term, one premature birth, 0 abortions, and three living children) woman at 26 weeks and 5 days gestation with history of combined peripartum and anthracycline-induced cardiomyopathy [previously left ventricular ejection fraction (LVEF) 10–15% and recently 40–45%] presented with acute decompensated heart failure. Her clinical status deteriorated with a drop in LVEF to 15–20% with a significant increase in pulmonary pressures and worsening mitral regurgitation. A multidisciplinary decision with the cardio-obstetrics team was made to place a pulmonary artery catheter for invasive haemodynamic monitoring and IABP insertion prior to delivery. Intra-aortic balloon pump support had a profound immediate decrease in her systemic and pulmonary vascular resistance allowing for a successful repeat caesarean delivery. Her haemodynamics remained stable after IABP removal and pulmonary pressures improved. She was discharged one week following her delivery on guideline-directed medical therapy.

**Discussion:**

Our case highlights the use of prophylactic intrapartum IABP in combined anthracycline-induced and PPCM and begins to explore its safety and efficacy in this high-risk patient population.

**Learning points**

- Subsequent pregnancies in patients with a history of peripartum cardiomyopathy (PPCM) are associated with a high risk of relapse with recurrent heart failure, deterioration of LV function, and maternal death.
- The use of IABP for short-term stabilization of haemodynamics or as part of early implementation of mechanical circulatory support prior to delivery may be a feasible strategy for the management of PPCM and prevention of decompensated heart failure.

## Introduction

Pregnancy bears significant changes in cardiovascular physiology including an increase in maternal blood volume, heart rate, and cardiac output that can precipitate deterioration of cardiac function among patients with peripartum cardiomyopathy (PPCM). Prior exposure to cardiotoxic cancer therapies may increase the risk of future PPCM.^1^ In this context, subsequent pregnancy in patients with a history of PPCM is associated with high rates of relapse, recurrence of heart failure, and maternal death.^2^ The management of PPCM remains a clinical challenge and few studies have explored the use of mechanical circulatory support (MCS) in PPCM within the unique haemodynamics of pregnancy.^3^ We present a case of early implementation of intra-aortic balloon pump (IABP) therapy for acute stabilization and intrapartum support in PPCM.

## Summary figure

Early use of intrapartum intra-aortic balloon pump support for haemodynamic stabilization of peripartum and anthracycline-induced cardiomyopathy: a timeline of events. Graphical timeline of events leading up to fourth pregnancy complicated by decompensated heart failure supported by insertion of IABP for stabilization and intrapartum support of repeat caesarean delivery culminating in uncomplicated delivery of mother and foetus.

**Figure:**
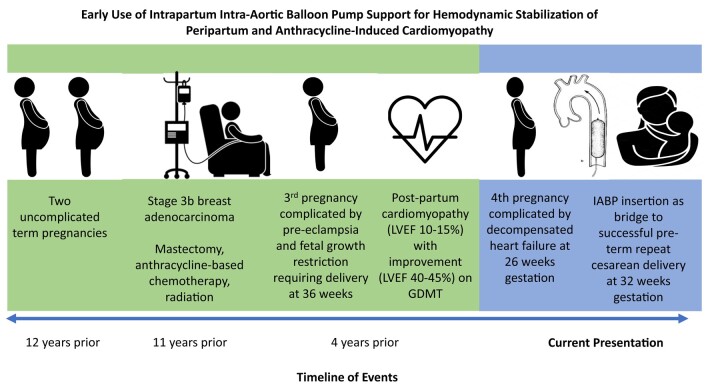


## Case presentation

A 36-year-old G4 P2103 (4th pregnancy, two full-term deliveries, one premature delivery, 0 abortions, and three living children) Black female at 26 weeks and 5 days gestation with a history of combined anthracycline-induced and PPCM presented with acute dyspnoea in hypoxaemic respiratory failure. Her past medical history was notable for hypertension, obesity (body mass index 46 kg/m^2^), and a diagnosis of Stage 3b breast adenocarcinoma 11 years prior, which required bilateral mastectomy, daunorubicin chemotherapy (cumulative dose unknown), and radiation therapy. Her pregnancy history was notable for two full-term pregnancies with spontaneous vaginal deliveries, and a third pregnancy 7 years after her breast cancer diagnosis complicated by pre-eclampsia requiring pre-term caesarean delivery. One month after her delivery, she was diagnosed with new cardiomyopathy most suggestive of PPCM [left ventricular ejection fraction (LVEF) 10–15%]. She had no prior history of cardiomyopathy following her breast cancer therapies. After her third pregnancy, she had recovery of LVEF to 40–45% on maximally tolerated guideline-directed medical therapy (GDMT). She reported discontinuing her heart failure medications (including losartan 50 mg daily, spironolactone 25 mg daily, and carvedilol 6.25 mg twice daily) upon discovery of her pregnancy status in early first trimester. Her outpatient cardiologist had discussed starting her on labetalol for management of hypertension in pregnancy, but this was deferred given normal blood pressures at the time. Soon after, she noted worsening dyspnoea and orthopnoea. On arrival to the emergency room, her exam was notable for heart rate 115, blood pressure 148/113 mmHg, respiratory rate 36 with an oxygen 2 saturation of 95% on 6 L high flow nasal cannula with jugular venous distension and bilateral pulmonary crackles. Brain natriuretic peptide was 350 pg/mL, and electrocardiogram was remarkable for sinus tachycardia with non-specific T wave inversions.

**Figure 1:**
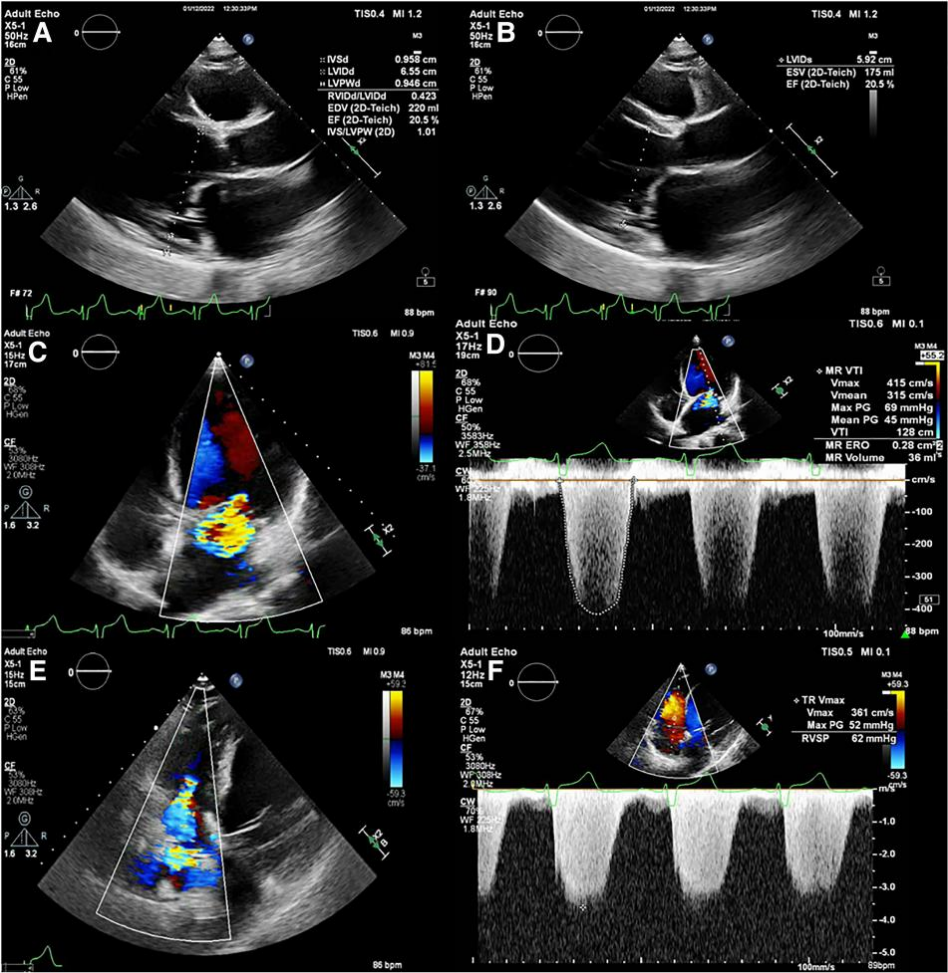
Non-invasive structural and functional echocardiographic assessment prior to IABP placement. LV systolic dysfunction and dilation in diastole (*A*) and systole (*B*). Moderate MR severity assessed by colour (*C*) and continuous flow Doppler (*D*). Moderate TR by colour Doppler (*E*) and peak tricuspid regurgitant velocity estimate of RVSP (*F*).

**Figure 2:**
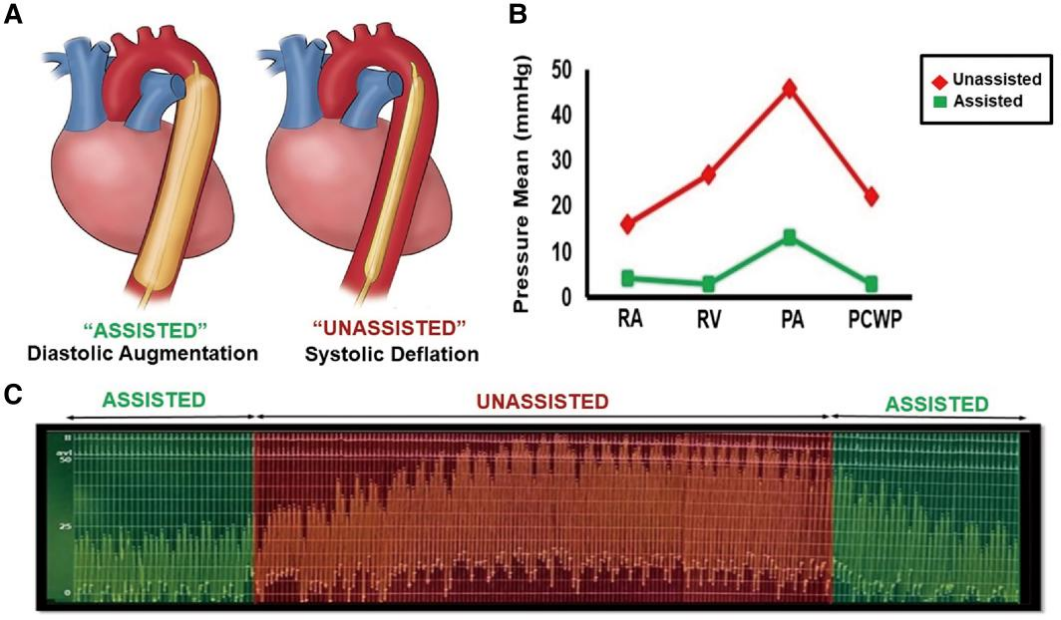
Invasive haemodynamics with the use of IABP. Graphical representation of IABP function with augmentation in diastole and afterload reduction in systole (*A*). Haemodynamic differences with and without IABP support for each parameter assessed (RA, RV, PA, PCWP) (*B*). RVSP in the presence (‘IABP assisted’) and absence (‘IABP unassisted’) of IABP support (*C*).

She was hospitalized for optimization of GDMT and serial foetal assessments under the interdisciplinary care of our cardio-obstetrics team. On admission, transthoracic echocardiogram revealed severe left ventricular (LV) dilation (left ventricular end diastolic dimension 64 mm) with LVEF of 15–20% with severe global hypokinesis, right ventricular systolic pressure (RVSP) of 46 mmHg, and mild tricuspid (TR) and mitral regurgitation (MR) (see Supplementary material online, *Video S1*). Her clinical status remained tenuous but stable through 32 weeks when she developed increasing signs of volume overload and oxygen requirement despite escalating diuretic therapy. Transthoracic echocardiogram demonstrated a significant increase in TR and MR severity with progressive elevation in estimated pulmonary pressures (RVSP 71 mmHg) suggestive of early decompensation of LV function (*Figure 1*) and (see Supplementary material online, *Video S2*). She was transferred to the cardiac intensive care unit, and a right heart catheterization was performed using minimal fluoroscopy and appropriate abdominal shielding, which demonstrated the following filling pressures: right atrium 16 mmHg, right ventricle (RV) 60/10, pulmonary artery pressure (PAP) 58/28 mean 46 mmHg, and pulmonary capillary wedge pressure (PCWP) 18 mmHg. After multidisciplinary discussion with the cardio-obstetrics team, the decision was made to place an IABP prior to delivery in the context of elevated filling pressures, primarily post-capillary pulmonary hypertension, and worsening TR and MR suggestive of progressive LV decompensation at the peak haemodynamic stress of pregnancy. This was chosen over inotropic support due to relative hypotension (systemic blood pressure 96/64, mean arterial pressure 74) and inability to tolerate GDMT. Intra-aortic balloon pump counter-pulsation support significantly aided in an immediate reduction and normalization of intracardiac pressures to a PCWP of 5 mmHg and mean PAP of 14 mmHg (*Figure 2*) as well as augmentation of cardiac indices (Fick cardiac output 5.9 L/min, cardiac index 2.7 L/min). With IABP insertion, the patient had a profound decrease in systemic (SVR) and pulmonary vasculature resistance (PVR) with an SVR of 1135 dynes s/cm^5^ and PVR 1.7 WU (Wood unit), allowing for a successful repeat caesarean delivery of a healthy male infant.

The IABP was effectively weaned on post-partum Day 3 without complications, and her haemodynamics remained stable after removal. Repeat echocardiogram demonstrated stable LVEF 20–25% with trivial MR and an RVSP of 26 mmHg. After having a risk and benefit discussion, the patient did not plan to breastfeed or express milk following delivery, which did not prompt the use of bromocriptine for her PPCM. She was discharged one week following her delivery on the following GDMT: carvedilol 15.625 mg twice daily, sacubitril-valsartan 49–51 mg twice daily, spironolactone 25 mg daily, dapagliflozin 10 mg daily, hydralazine 25 mg twice daily, isosorbide mononitrate 60 mg daily, and apixaban 5 mg twice daily for LV thrombus prophylaxis. Post-partum cardiac magnetic resonance imaging demonstrated a mild improvement in LVEF to 34% with no late gadolinium enhancement or LV apical thrombus but persistently dilated LV cavity (indexed LV end diastolic volume 128.51 m^2^; LV end systolic volume 85.07 m^2^). The patient continues to do well with New York Heart Association class I–II symptoms and has only been hospitalized once for heart failure (in the context of cholelithiasis). She is undergoing bariatric surgery assessment before being considered for future advanced therapies including heart transplantation.

## Discussion

Cardiovascular disease is the leading cause of maternal death (up to 12% due to cardiomyopathy) in the first year following pregnancy, and women with a history of prior PPCM with non-recovered LVEF are at significantly high risk during subsequent pregnancies.^2^ Risk factors for PPCM include Black race, advanced maternal age, hypertension, pre-eclampsia, and multiple gestations,^4^ most of which were relevant in our patient. Some animal studies show a potential association between elevated prolactin levels and PPCM, though further studies are required in human subjects.^4^ Cardiogenic shock (CS) complicating the intrapartum period is rare but is associated with a high mortality rate. The use of inotropic support is widely accepted for the treatment of CS in non-pregnant patients but has not been well studied in PPCM.^5^ Instead, MCS initiation including IABP, percutaneous LV assist devices, and extracorporeal membrane oxygenation has been used with some success among this population.^6^ While afterload reduction is a mainstay in the management of decompensated heart failure, counter-pulsation can augment cardiac output by reducing afterload without substantially lowering the mean arterial pressure. Prior studies have identified unique characteristics of ‘hyper-responders’ to IABP therapy. Elevated pulmonary artery pressures, high SVR, and the presence of at least moderate MR are key predictors of early haemodynamic response to IABP therapy among non-pregnant individuals (all of which were present features in our patient).^7^ Intra-aortic balloon pump in PPCM has primarily been used for the post-partum management of CS as a salvage mechanism or bridge to advanced therapies.^8^ Interestingly, IABPs have been used as a prophylactic measure pre-cardiac surgery with improved outcomes.^9^ However, to date, there have been limited reports of the prophylactic use of IABP to assist with intrapartum delivery, or predictors of super-responders to this therapy. Our case highlights the use of prophylactic IABP in combined anthracycline-induced and PPCM demonstrating a super-responder to this therapy and begins to explore its safety and efficacy among this high-risk patient population. However, it should be noted that it is important to continue to make individualized case decisions regarding the appropriate patient who may benefit from MCS. Thus, the use of MCS in pregnancy, particularly, the early use of IABP to support delivery, still requires ongoing research for optimization of candidate selection and assessment of short- and long-term maternal and foetal outcomes.

## Lead author biography


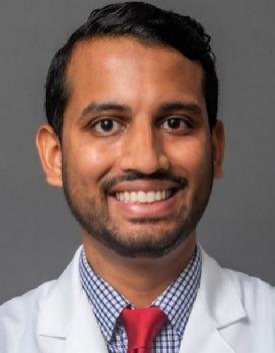
 Dr Karthic Chandran is an interventional and structural cardiology fellow at the University of South Florida Morsani College of Medicine in Tampa, Florida. His current interests lie with valvular cardiomyopathy and cardiogenic shock treatment with mechanical circulatory support devices.

## Supplementary material

Supplementary material is available at *European Heart Journal – Case Reports* online.

## Supplementary Material

ytae033_Supplementary_Data

## Data Availability

The data underlying this article will be shared on reasonable request to the corresponding author.
